# Mental health in Brazil in times of neoliberalism and pandemic: retrogression and resistance

**DOI:** 10.1590/S0104-59702024000100017en

**Published:** 2024-05-17

**Authors:** Cláudia Freitas de Oliveira

**Affiliations:** i Professor, Department of History/Universidade Federal do Ceará. Fortaleza – CE – Brazil claudia.oliveira@ufc.br

**Keywords:** Mental health, Psychiatric reform, Neoliberalism, Pandemic, Resistance, Saúde mental, Reforma psiquiátrica, Neoliberalismo, Pandemia, Resistências

## Abstract

This study analyzes aspects of mental health in Brazil as an active political field involving a range of social segments and actors from opposing fields in a context of advancing neoliberalism and pandemic. The analysis begins in 2016, when fiscal austerity entered the national agenda, and proceeds through the pandemic until the present day, when both phenomena continue to prevail, even if the intensity of the pandemic is now reduced. In the ambit of mental health, the national policy based on the principles of the psychiatric reform has suffered severe setbacks. Nonetheless, despite state-sponsored efforts to discourage social control and public participation, important sectors of society are engaged in active resistance.

## Resumo

O trabalho analisa aspectos da saúde mental no Brasil como campo político ativo, que envolve vários atores e segmentos sociais em áreas de disputa opostas, em tempos de pandemia e de avanço do projeto neoliberal. Tem como início de delimitação temporal 2016, quando as políticas de austeridade fiscal entram na agenda nacional, e vai até os dias atuais com o cenário da pandemia, ambas ainda vigentes, embora a pandemia tenha diminuído sua intensidade. No âmbito da saúde mental, a política nacional enfrenta profundos retrocessos quanto aos princípios da reforma psiquiátrica. Entretanto, a despeito da tentativa do Estado de desestimular o controle social e a participação popular, observam-se resistências de importantes setores.

This article seeks to contribute to the historiographical debate by reflecting on aspects of recent Brazilian history through a coordinated and dialogic documental and literature review of mental health in the context of the pandemic and the neoliberal project underway in the country. In 1921, the foreign press, drawing on data analyzed by the World Health Organization, reported that Brazil was the world’s biggest open-air covid-19 laboratory in the world. The data pointed to a serious public health threat not only for the Brazilian population – above all, the poorest, verging on abject poverty – but for Latin America and around the world. In a news story published in the Brazilian Academy of Science (Academia Brasileira de Ciências, ABC) on March 4, 2021, entitled “Brazil is set to become the biggest biological reservoir of the coronavirus in the world” (“Brasil vai se transformar no maior reservatório biológico do coronavírus no mundo”), based on an interview given to the British newspaper *The Guardian*, the neuroscientist and member of the ABC Miguel Nicolelis is quoted as saying that the Brazilian government’s actions in fighting the pandemic represented an international risk. It was not, therefore, a matter that was restricted to the country’s borders: “This is about the world. It’s global,” warned the scientist (ABC, 4 mar. 2021).

In this context, many political and scientific analyses published in specialized publications and the mainstream media demonstrated unequivocally that the mismanagement of the pandemic by the Jair Bolsonaro government, whether by intent or through negligence or omission, was instrumental in the deterioration of the public health crisis. The shocking scenes in Manaus (Falta de oxigênio..., 4 maio 2021; Muniz, 2021) in early 2021, when the hospitals ran out of oxygen, went down in the annals of Brazilian public health as one of its darkest chapters. This cannot be forgotten; indeed, it should be remembered and analyzed by future generations from a social and historical perspective.

Nonetheless, while the pandemic was undoubtedly an unprecedented episode for today’s generations, insofar as nobody in Brazilian society, even the oldest, had experienced anything quite so disruptive to the everyday life of men and women, there is another phenomenon, all too familiar to the Brazilian people, that cannot be ignored if we are to understand the full dimension of the tragedy foretold in the country: the policy of economic austerity that has exacerbated poverty to the extreme in Brazil in recent years. To understand the drama of the pandemic experienced by millions of Brazilians since 2020, it is crucial to analyze the austerity policies introduced before this, which have had a direct impact on the country’s most socioeconomically vulnerable populations.

## Constitutional amendment n.95 and austerity politics in Brazil: the hunger map and the Brazilian public health system

A serious economic recession accompanied by a major institutional political crisis in Brazil turned worse when constitutional amendment n.95 (PEC 241/2016) passed on December 2016 (Brasil, 15 dez. 2016), under the administration of Michel Temer. The amendment introduced a new fiscal regime, imposing a ceiling on primary expenditure based on inflation-adjusted spending in the previous year. The new fiscal regime froze the primary expenditure of the Union for 20 years, which, according to a prognosis published by the Institute of Applied Economic Research (Instituto de Pesquisa Econômica Aplicada, Ipea), was a cause for concern. In the words of the economist Manoel Pires (2016, p.133), “no country, even in a worse state than Brazil, has adopted a fiscal rule that has such a great impact on the management of the budget.”

Building on the approval by Congress of this constitutional amendment, the president who succeeded Temer, Jair Bolsonaro, made a point of consolidating the draconian policy of fiscal austerity from the moment he came into office, attacking above all the most sensitive areas affecting the population: education and health. In this perspective, by adjusting the public accounts – under the allegation it was the single most effective measure for overcoming the economic crisis – and, crucially, meeting the demands of the financial markets and neoliberal ideals, the Brazilian government progressively squeezed the budgets for social areas, diminishing the size of the state and thereby debilitating social policies.

However, in academic and social circles, opinions on the validity of the measures announced and introduced by the government to limit any increase in public spending and consequently enable economic development are anything but consensual. Researchers critical of the fiscal austerity policy make quite the opposite point: that the need for measures to encourage state spending on social areas is greatest at times of economic recession, because they have the effect of promoting social justice and future growth by investing in youth and the potential for growth of human capital. According to the researchers Isabela Santos and Fabíola Vieira (2018, p.2307), Brazil is still one of the countries with “the biggest social and income inequality in the world. … Despite this situation, the austerity agenda has been implemented in the country at great length in recent years.”

It should be noted that if the government’s discourse, aligned with the interests and guidance of large international financial entities, promotes the precepts of neoliberal fiscal austerity, in political terms it is deliberately biased in the fight to balance the public accounts. While it pursues a form of “selective austerity” that ends up penalizing the masses, it preserves, protects, and ensures the growth of earnings amongst the richest sectors of the country (Santos, Vieira, 2018, p.2311).

The fiscal adjustment policy, which hits the Brazilian population hardest, was not limited to 2016. The following year, continuing the neoliberal project and the financial crisis that had taken root since 2008, a labor reform was passed in Congress, followed, in 2019, by a social security reform.

In the current phase of contemporary global capitalism, the state is turning social policies into marketable products, whether it be through the privatization of state-owned companies or the conversion of public real estate into private property. The impacts on economic and social life have been felt sharply in Brazil, with an upsurge in the unemployment and poverty figures, exacerbating an already precarious status quo along with the potential for social disruption.

In 2020, when the covid-19 pandemic broke out, no efforts were made to rein in the neoliberal policy that prevailed around the world. Quite the contrary: states intensified bailouts for big market and capital players, while populations languished in deepening poverty and workers faced increasingly precarious conditions. “Never before has the economic action of states been so instrumental in allowing the satanic bourgeois treadmill to crush the life and rights of the working class” (Granemann, Miranda, 2020, p.27).

In Brazil, things were no different. Attacks on the working classes intensified even in the first year of the pandemic. While maintaining its fiscal austerity measures, the federal government – the mainstay of and proxy for the financial and economic elites – announced its political plans for handling the pandemic. Even in 2020, cries of genocide being carried out against the Brazilian population started to enter the political agenda and lexicon.

It is fair to state from the way the covid-19 health policy developed in Brazil that genocide is underway, responsibility for which lies at the door of the leaders of different Brazilian state entities and the haute bourgeoisie; neither one nor the other seems unduly perturbed by the lethal nature of the disease or the speed with which it is spreading, as long as the bourgeois state can pave, with public monies, a way out for its – and big capital’s – economic crisis (Granemann, Miranda, 2020, p.32).

As such, the perverse effects of the prevailing neoliberal policy grew to such an extent that, in 2020, as social inequality became increasingly exacerbated, Brazil was put back on the Hunger Map (Rede Penssan, 2021). This was the first time it had appeared on the map since it was removed in 2014, in response to a political agenda that had put great stock on tackling hunger. At the time, it was reported that the “undernourishment rate in Brazil fell by half from 10.7 percent to below 5 percent” in the global reports published by the Food and Agriculture Organization of the United Nations (FAO, 2014, p.23).

Constitutional amendment n.95 and the pandemic, compounding the consequences of the neoliberal policies embedded by two federal administrations, produced effects never seen in the history of Brazil. In terms of health, Ipea predicted that the constitutional amendment would bring about a reduction of “743 billion reais in 20 years for the funding of the public health system and for assuring the right to health of the most vulnerable social groups” (Guerra, Bezerra, Carnut, 2020, p.1239).

In the area of health, the damage caused by the new fiscal regime directly affected the public health system, Sistema Único de Saúde (SUS), which, as set forth in the 1988 Constitution, is mandated to provide universal, comprehensive, equitable access to goods and services. Since the austerity policy was introduced, SUS has gradually faced not only underfunding, but an actual erosion of its state funding. Besides weakening its ability to provide universal access to health and engage in public policymaking, this also promotes flexibilization and encourages a shift to supplementary (private) healthcare, meeting the demands of private healthcare market players. Constitutional amendment n.95, with its focus exclusively on fiscal issues, fails to take account – intentionally or not – of a host of social, physical, and economic factors and elements, from the ageing of the population to the judicialization of healthcare and the need for the introduction of new technologies (Sá, 2018, p.9).

The prognoses published in reports and technical briefs by economists and public policy and public administration experts from Ipea before the covid-19 pandemic already predicted a real negative impact on the provision of healthcare and the structural integrity of SUS. It is important to note, however, that even during the pandemic, the fiscal policy and the constitutional amendment were not put on the agenda for revision by the Brazilian Executive or Legislative branches. Rather, as the country’s social and public health problems intensified, the levels of social inequality were exacerbated to an unprecedented level. One area of health that was severely impacted was mental health, which, for its singularities, deserves to be considered as a public policy in its own right. To understand it in contemporary times, we must first problematize the historic trajectory of public clashes and struggles with market interests.

## A brief history of mental health: between public policies and market interests

In 2016, the neoliberal project pursued by the administrations that came to power in the wake of the toppling of President Dilma Rousseff dealt mental health in Brazil a major blow. Not only did mental healthcare suffer under the fiscal adjustment because of the underfunding and “defunding” of the Psychosocial Care Network (Rede de Atenção Psicossocial), but the area also came under attack by policies designed to roll back more recent advances in the area, reintroducing many ideas whose roots could be traced to the civil and military dictatorship in the country, when the “insanity industry” was encouraged and sponsored by the government, to the benefit of the private medical and hospital sector.

In 1977, the National Institute of Social Security (Instituto Nacional de Previdência Social) implemented a “psychiatry plan,” which aimed to “promote the modernization of care in the field of mental health.” According to the public health specialist and advocate for the public health reform at the time, Carlos Gentille Mello (1981, p.99), “the results were apparently paradoxical. Social Security was forced to pay the 351 hospitals contracted no less than one billion additional cruzeiros, corresponding to 200,000 unnecessary hospitalizations.”

In 1978, ten medical entities from Rio de Janeiro sent a manifesto to the minister for health, Paulo de Almeida Machado, claiming that

‘the existing mode of psychiatric care underway is ineffective, chronifying, and elitist.’ And that ‘this distortion enables a veritable insanity industry to flourish, constituted of huge hospitals, which, with the eternal rehospitalization of mental patients, made chronic, has a never-ending source of profit, funded mainly by the Social Security’ (Mello, 1981, p.113).

Thanks to a political environment marked by denouncements and resistance on the part of democratic segments of mental healthcare, the 1970s saw the beginnings of a heterogeneous movement involving representatives from a range of civil society sectors. Known as the psychiatric reform, the movement was part of a broader move towards a return to political democracy after a long period of civil-military rule and was initially linked to the public health reform in the struggle for the universalization of the right to health, whose roots could be traced back to the First Republic (1889-1930). In this period, health became a political and social issue as it was transformed into a collective good through the expansion of state authority throughout the nation, upon which the foundations for the creation of a mental health system were based, marked by the development of plans to organize Brazilian nationhood through the presence of the state, as well as the centralization and verticalization of the central government (Lima, 2005, p.39). In the 1970s, the public health reform, which contained certain tensions and contradictions within the broader Brazilian public health movement, enabled the emergence of the psychiatric reform, which spoke out not only against the “insanity industry,” but also against the violence and other asylum-inspired practices inside psychiatric hospitals. Under the banner of the reforms, the movement proposed a new model for mental healthcare rooted in the principle of psychosocial rehabilitation and care in a broader context of deinstitutionalization.

The psychiatric reform galvanized actions in different spheres with a wide range of strategies: in the press, which published stories denouncing rights violations in psychiatric hospitals; in academia, with scholarly discussions among specialists in a range of knowledge domains; and even in the political arena, in which the professionals working at psychiatric hospitals and mental health service users and their relatives engaged in acts of resistance. As such, an array of political and legal tactics was employed to put pressure on Congress to pass the new pro-deinstitutionalization mental health legislation (Lougon, 2006; Oliveira, 2020; Paulin, Turato, 2004; Wadi, Olinto, Casagrande, 2015; Yasui, 2006).

Although these political and legal efforts mobilized progressive sectors of society, they were countered by the actions of conservatives, particularly some key mental health business players, who also put strong pressure on Congress to maintain the hospital-centered psychiatric order and the existing dependence on institutionalization in mental asylums. Only in 2001, after more than ten years of discussions in the federal legislature, was the bill that enacted the psychiatric reform passed (Amarante, 1995; Amarante, Nunes, 2018; Pereira, 2004).

Resistance by organized civil society was crucial in getting the new law (n.10.216/2001) passed. Nevertheless, medical and business entities that felt hard done by in the reform never ceased to exert pressure on the federal executive and legislative branches to increase the number of psychiatric beds. When, in 2016, the progressive government of President Dilma Rousseff was toppled by the combined institutional action of Congress and the judiciary, conservative and pro-privatization segments of mental health saw it as an opportune moment to once again push their agenda for the national mental health policy. And that is what they did. Nonetheless, it should be noted that therapeutic communities were already included in the Psychosocial Care Network in 2011, during the Rousseff administration, through directive n.3.088, of December 26, as a ramification of the vigorous endeavors of the anti-crack lobby in Congress (Brasil, 23 dez. 2011).

## Mental health in contemporary Brazil: retrograde steps and acts of resistance

In 2017, during the administration of Michel Temer, a new national mental health policy was taking shape nationally, as the Ministry of Health published its first normative acts, directly countering the principles and guidelines of the psychiatric reform, as per law n.10.216. These publications immediately roused organized civil society against this new trend, since the ministerial changes were imposed with no broad-based or public consultation with different segments, such as social movements, boards of health, psychosocial care workers, or the users of public mental health services and their families.

On September 6, 2017, a meeting was held by the National Council of State Secretaries of Health and the National Council of Municipal Secretaries of Health that prompted the Brazilian Association of Collective Health (Associação Brasileira de Saúde Coletiva, Abrasco) to publish a note in defense of the psychiatric reform, calling for a dignified and contemporary mental health policy. At the aforementioned meeting, a proposal had been put forward to alter the way mental health care was delivered, “expanding psychiatric hospitals in a context of the freezing of investments in the public health system for twenty years.” Abrasco (6 set. 2017) described this proposal as “obscurantist, retrograde, and anti-scientific.”

On December 11, Abrasco spoke out against the new mental health policy that was being pushed for primarily by the General Coordination of Mental Health, Alcohol, and Drugs of the Ministry of Health, describing it as being grounded in an “asylumization approach.” In a new release, the association stated that the current asylum models, represented by therapeutic communities, treated mental health service users as “people with mental disorders and not people with political and citizens’ rights.” Institutionalization was not only reductionist, but also promoted the “increased medicalization and commercialization of life” (Abrasco, 11 dez. 2017).

Therapeutic communities reproduce in contemporary times the basic hallmarks of the mental asylums of the nineteenth and twentieth centuries. In other words, they are places where users are kept in hermetic environments, isolated from the outside world, violating patients’ human rights and reviving the same stigmas of old, insofar as the community members are treated as “unruly and undisciplined.” The organization of therapeutic communities is grounded in strict discipline, plus a considerable dose of religious doctrine, particularly of a Pentecostal order, whose beliefs “predominate in every macro region of the country.” According to a release by Ipea, “the vast majority of Brazil’s therapeutic communities are linked to churches and religious organizations (82%), primarily Christian” (Ipea, mar. 2017, p.21). Therapeutic communities rooted in the “three pillars of work, discipline and spirituality” (p.35) were given new impetus under the Bolsonaro government, with its particular political and ideological slant, which resulted in the intensification of policies designed at evangelization in the field of mental health. One salutary example is the use of sexual reorientation therapy, which has no scientific, ethical, or legal basis (Simon, 2020, p.19).

Notwithstanding the work by several sectors of society to draw attention to the attacks being made on the psychiatric reform, the offensive by the Brazilian state was undeterred. On December 14, 2017, the Ministry of Health published resolution n.32, in which it set new guidelines for the Psychosocial Care Network, introducing mental health outpatient clinics and psychiatric hospital beds to the network without previously engaging in extensive discussions with professionals from the area or the social actors who occupy the spaces of social control (Brasil, 14 dez. 2017). On December 21, a directive n.3.588 was published (Brasil, 21 dez. 2017) that amended the previous directives and significantly weakened the Psychosocial Care Network. It was not extinguished, but it was debilitated insofar as the new directive made psychiatric hospitals the main beneficiaries of financial investments. It is important to note that despite enthusiastically advocating austerity in healthcare, the Brazilian state channeled significant funding towards owners of therapeutic communities while continuing to underfund the Psychosocial Care Network. In August 2018, the Ministry of Health published directive n.2.434, “in which it readjusted the value of the daily rate for hospital-based care over 90 days and encouraged hospitalization,” bowing to the pressure of the lobby of psychiatric establishments and physicians (Brasil, 15 ago. 2018).

Entities representing the sector spoke out against the dismantling of public mental healthcare, and in particular the work of the Brazilian Association of Psychiatry (Associação Brasileira de Psiquiatria), one of the federal government’s foremost allies in the push for hospital-based psychiatric care. In September 2018, the 15th region of the Regional Council of Psychology (Conselho Regional de Psicologia, CRP), in Alagoas state, published a repudiation of the association’s position in response to a message it had published alluding to the “yellow September campaign,” in which it had stated that “suicide [is] a medical emergency” and that “just talking does not fix it.” In a sharply worded response, the Alagoas branch of the CRP wrote:

It is our understanding that this statement is evidence of the Brazilian Association of Psychiatry’s commitment to corporate and market interests, turning the complex subject of human suffering into a niche to be capitalized upon. This does not surprise us, however, because the institution in question has taken a deliberately favorable stance towards the retrogression seen in the national policy of mental health, which directs public money towards private institutions that operate as mental asylums, to the detriment of investments in the expansion of public community services like the Centers for Psychosocial Care (CRP-AL, set. 2018).

Resistance to the political and therapeutic retrogression in the area of mental health took various guises. It was not expressed only by entities representing professionals from related areas, but also mobilized groups and organizations from academia and social movements. In September 2018, Abrasco submitted a public request to the Brazilian state and a private request to the Mental Health Coordination of the Ministry of Health and the National Secretariat for Drugs Policies of the Ministry of Justice asking for assurances of transparency and access to data and indicators on mental health in the Brazilian population. Information of this nature – fundamental for fostering and developing public policies in the area – had ceased to be published in 2016 (Abrasco, 24 set. 2018). In parallel, in November, the Ministry of Health issued the directive n.3659 (Brasil, 14 nov. 2018) suspending all funding for the services offered through the Psychosocial Care Network.

Also in November 2018, dozens of groups took a public stand against the creation of the mixed parliamentary front in defense of the new national policy for mental health and hospital-based psychiatric care, which, under the guise of developing a “new mental health policy,” planned to revert to the asylum model that had preceded the public health reform and the creation of SUS (the public health system) in the 1980s. The action taken by a broad range of social actors resulted in the launch of the mixed parliamentary front in defense of psychiatric reform and the fight against asylums (Brasil, 15 maio 2019).

In 2019, as a result of significant engagement over the previous years by civil society and public officials, through the mediation of the Federal Public Prosecution Service (Ministério Público Federal), the Federal Public Prosecution Service for Citizens’ Rights (Procuradoria Federal dos Direitos do Cidadão), the Federal Council of Psychology (Conselho Federal de Psicologia), and the National Mechanism for the Prevention and Combat of Torture (Mecanismo Nacional de Prevenção e Combate à Tortura), a report was published entitled Psychiatric Hospitals in Brazil: Report on a National Inspection (“Hospitais Psiquiátricos no Brasil: Relatório de Inspeção Nacional”). The document details the dramatic and inhumane situation within Brazil’s psychiatric hospitals and clinics (CFP et al., 2019).

The report was the result of inspections of forty psychiatric institutions in different states of Brazil, which revealed unequivocal evidence of rights violations. After the presentation and description of the structural and human conditions encountered in the establishments, the report recommends actions to be taken by a range of public entities, including the need to “close the front door, so that no new hospitalizations occur, which will enable the first steps to be taken towards the planned discharge of all those people in these institutions, with a view to their deinstitutionalization, in compliance with the law” (Brasil, 12 dez. 2019, p.505). The report stresses the need for the deinstitutionalization of all people hospitalized in these establishments and sets forth several procedures to be taken by executive bodies and the hospital management teams.

Also in 2019, the National Internuclear Anti-Asylum Network (Rede Nacional Internúcleos da Luta Antimanicomial, Renila), representing groups and movements advocating for the end of asylums in different Brazilian states, published a public manifesto repudiating the new national policy for mental health, alcohol, and other drugs proposed by the Ministry of Health, presented in both directive n.3.588/2017 and technical note n.11/2019 (Brasil, 4 fev. 2019). One of the measures proposed in the technical note was the encouragement of the use of electroconvulsive therapy, which was added to the national list of permanent equipment and material that could be funded via SUS, for use in general and specialized hospitals (Brasil, 4 fev. 2019).

In this document, Renila calls the use of electroconvulsive therapy “a controversial practice, to say the least, and absolutely invasive.” It condemns “the hospitalization of children and adolescents, who need and have the special right to be treated in freedom, with dignity and protection, together with their family and in the community.” It further criticizes the priority the Ministry of Health places on financing psychiatric hospitals and therapeutic communities and “invest[ing] financially in resources that violate rights and recreat[ing] obsolete and iatrogenic services” (Renila, 27 fev. 2019).

The manifesto ends by reiterating the principles of the Brazilian psychiatric reform:

The fight against asylums firmly sustains its ideals and radically defends the Brazilian psychiatric reform and the Brazilian public health system. It will not accept retrogressions and will not turn its back on the assurance and realization of citizens’ rights and the conditions for the expression and inclusion in the social fabric of the subjectivity of people in mental suffering and making abusive use of alcohol and other drugs (CRESS-MG, 25 mar. 2019).

The document represented the position of groups and entities against the introduction and entrenchment of the new mental health policy. Towards the end of the year, the Ministry of Citizenship published a call for tender to select therapeutic communities. Its goal was to expand the number of places for substance users in treatment in the country from 11,000 to 20,000. The estimate was that public funding for the private sector would increase from 153.7 million reais in 2019 to 300 million reais in 2020 (Brasil, 12 dez. 2019).

Brazil’s mental health policy, which had already been eroded as of 2017 – a trend that was exacerbated in 2019, when Jair Bolsonaro, a far-right politician, became president – took a serious turn for the worse in 2020, with the onset of the covid-19 pandemic. Indeed, the Bolsonaro government showed the country that its progress towards democracy was anything but complete. With top-ranking military personnel holding positions of leadership in the government, the establishment of a new bespoke information system that defied the control of republican entities, not to mention a whole raft of regulations and rules undermining democracy, the Bolsonaro project walked a fine line of constant tension between the military dictatorship past and a future perpetrated by fascist political action (Schurster, Gherman, 2020; Schurster, Silva, 2021). The government, politically authoritarian and economically neoliberal, fueled the neglect and deterioration already prevailing in several sectors of society, including mental health, as part of the “new” mental health policy. There was an intensification of the rationale supporting the normalization of institutionalization, which reshapes the lives of people who experience such confinement and are abandoned and “left to die,” condemning them to the conditions and “zone of social neglect” (Biehl, 2008, p.439).

In a specific scenario, from a global perspective, the pandemic revealed the extremely fragile nature of public health systems and highlighted the “neocolonial nature of global health,” whose responses, underpinned by the neoliberal capitalist order, fulfilled the geoeconomic interests of the private sector. The pandemic further highlighted a structural vulnerability, in that it disproportionately affected “underrepresented groups from the social strata” (Biehl, 2021, p.343).

## Mental health in Brazil and the covid-19 pandemic

According to Joel Birman (2020), the pandemic had an unequivocal influence on mental health, both in terms of its effects on individuals’ mental wellbeing and also in the more general increase in stress experienced worldwide. Despair and despondency were some of the mental health consequences of the pandemic, seen not only as a biological and public health phenomenon, but also for its undeniable effects on the economy, politics, and culture, therefore requiring an interdisciplinary perspective to be understood. This is because the virus invaded people’s lives as an “intangible, invisible enemy,” which prompted widespread “terror of death” in individual psyches, “setting the stage for psychic helplessness” (p.104). People felt they were at the mercy of chance, indetermination, or despair. For Birman, the pandemic can be understood as a combination of “catastrophe” and “trauma,” bringing about a psychic experience marked by a “traumatic infrastructure” arranged around different “symptomatic formations” (p.199).

As for the mental health measures taken by the government, the Ministry of Citizenship published the directive n.340/2020, on March 30, 2020, which established “measures for tackling the Nationwide Public Health Emergency arising from human infection by the novel coronavirus (covid-19) in the ambit of therapeutic communities” (Brasil, 30 mar. 2020a). The directive required therapeutic communities to continue to operate and even to admit new members during the pandemic. The measure prompted concerns amongst progressive social sectors. There were fears and even claims of forced internments, especially of drug users. Another worrying measure announced in the directive concerned inspection. Inspections should be made through online systems that the therapeutic communities themselves should fill out, as well as via telephone conversations. In other words, in practice, there was no longer any external or impartial oversight of the health and epidemiological conditions inside the therapeutic communities, which generally did not have the means to ensure social distancing among their members. At the beginning of the pandemic, alarming rates of infection were found amongst them. In May 2020, 36 of the 77 confirmed cases in Jaci, a municipality in Sao Paulo, were in therapeutic communities (Fabio, 25 jun. 2020).

During the pandemic, the logic of confinement in psychiatric institutions was reinforced. On March 30, 2020, the National Committee for the Prevention and Combat of Torture (Comitê Nacional de Prevenção e Combate à Tortura), the National Mechanism for the Prevention and Combat of Torture (Mecanismo Nacional de Prevenção e Combate à Tortura), and the National Council for Human Rights (Conselho Nacional dos Direitos Humanos) published a joint release in which they manifested concern about the covid-19 pandemic, especially to specific cases, such as people who had been deprived of their liberty and in long-stay institutions, such as psychiatric hospitals and therapeutic communities (Brasil, 30 mar. 2020b). The reason was that these institutions had historically been insalubrious, overcrowded, and the target of limited welfare policies. As the pandemic worsened, they could not observe the minimum sanitary guidelines required to contain the spread of the virus.

In May 2020, Renila published a document on “the Brazilian government’s necropolitics and drugs policy under the pandemic.” Undersigned by dozens of associations, forums, commissions, and other groups, the document called for directive n.340/2020 to be revoked immediately. The text stated that the fifth article of this directive infringed citizens’ rights when it stated that “admissions, once made, should not be interrupted” (Renila, 3 maio 2020), because it ignored the will of the person regarding their care. In alignment with this text, the Minas Gerais branch of the Regional Council of Psychology (CRP-MG, 14 maio 2020) questioned whether the Ministry of Citizenship

disregards the fact that most of the lodgings in therapeutic communities are no more than a collection of beds occupied by a collection of people who can scarcely move around the cramped space? Do they show disregard for the risk of contagion associated with this lethal proximity because they also believe that Covid-19 is nothing but ‘a bit of a cold’ and that the trade in bodies and souls should not be interrupted?

According to the Renila publication, the directive attended to the “market clamor” of those who supported “institutions of segregation and violence” and who “reaffirmed the necropolitics of the present Brazilian government” (Renila, 3 maio 2020). Finally, according to CRP-MG (14 maio 2020), the directive

is gravely mistaken from a public health perspective, not contemplating a reduction in the number of institutionalized people, which would prevent a mass loss of life, and does not even propose the use of individual protective equipment or the requirement for a contingency plan to fight the novel coronavirus.

In August 2020, Brazil hit the milestone of hundred thousand people killed by the coronavirus – a number that could have been prevented if there had been a minimally competent and engaged management of the situation. However, scientific denialism and the “notably anti-Enlightenment hallmark” of the government, driven politically and programmatically by a neo-Pentecostal discourse, relaxed the social distancing measures. The social inequalities already produced, the neoliberal order, and the “radical experiment in normative discontinuity brought about by the pandemic” intensified the inequities and disparities (Birman, 2020, p.72), leading to a policy geared not towards the most needy in the population, but towards serving the rationale of the market and profit, feeding the already swollen coffers of the pharmaceutical industry in general and the psychopharmaceutical industry in particular.

The pandemic accentuated the socioeconomic, racial, and gender-based inequalities already exacerbated in recent years. Specifically in the arena of mental health, it brought about heightened psychosocial demands. With covid-19, the far-right government did nothing to restrain the attacks on the mental health policy rooted in the psychiatric reform. On the contrary, it promoted a psychiatric counterreform, renewing the asylum-based, private-sector model.

Yet if the rollback and debilitation of Brazil’s mental health policies and mental healthcare for the population was latent, it also brought about coordination and reaction on the part of a range of social segments throughout the country willing to fight back.

## Resistance during the pandemic

With the conjunction of two tragic realities – the pandemic (within the broader health context) and the psychiatric counterreform (of mental health in particular), several segments of society started to speak out and fight against the dismantling of the country’s public mental health policies. The end of 2020 was particularly fraught when it came to the defense of inclusive, accessible, and democratic mental health care.

On December 4, the country’s conservative political and economic forces turned their energies towards imposing a psychiatric counterreform policy, which was undertaken by the federal government with the direct support of the private sector. This took place during a meeting of the National Council of State Secretaries, when representatives from the Ministry of Health proposed revoking directives from 1991 to 2014 that underpinned the existing national mental health policy. This proposal was substantiated by a document submitted by the Brazilian Association of Psychiatry and signed by entities such as the Brazilian Medical Association (Associação Médica Brasileira), the Federal Council of Medicine (Conselho Federal de Medicina), the National Federation of Physicians (Federação Nacional dos Médicos), the Brazilian Association of Impulsivity and Dual Pathology (Associação Brasileira de Impulsividade e Patologia Dual), and the Brazilian Society of Neuropathology (Sociedade Brasileira de Neuropsicologia) (ABP, 2020).

The Brazilian Association of Psychiatry’s document, entitled Guidelines for a Model for Comprehensive Mental Health Care in Brazil 2020 (“Diretrizes para um modelo de atenção integral em saúde mental no Brasil 2020”), was one of the most audacious attacks by conservative and private players against public policies for mental health in Brazil, insofar as it called for the extinction of a range of therapeutic and social actions and of programs for the social rehabilitation of people with mental disorders, such as the “back home” program, as well as services for the most vulnerable populations, such as homeless people, ending the mobile care teams (ABP, 2020).

In the face of a concrete threat against decades of struggle and progress by the anti-institutionalization movement encapsulated in the psychiatric reform, based on the pillars of valuing, and assuring the human rights of people with mental disorders and in psychic suffering, organized civil society took action. Representative groups from the areas directly affected, professionals from a range of disciplines, service users and their families, as well as members of Congress, were quick to galvanize and kick off a counterreaction in response to the attempt by actors from the public and private sectors to revoke these key directives and adopt a biomedical and segregating institutional model.

Alongside the anti-institutionalization movements that had operated historically in Brazil, like Renila, new networks were formed, such as the United Front for the Defense of Mental Health, Psychiatric Reform, and Anti-asylum Struggle (Frente Ampliada em Defesa da Saúde Mental, da Reforma Psiquiátrica da Luta Antimanicomial, Fasm),

On December 4, the first meeting was held, using an online platform,^1^ which was attended by over 250 people. The agenda was: to draft a letter repudiating the document published by the Brazilian Association of Psychiatry – one of the key players in the psychiatric counterreform; to set up working groups to optimize the different proposed interventions; to arrange a general meeting in the defense of mental health; and to discuss the creation of a general conference on mental health.

Intense behind-the-scenes activity resulted in the participation of 3400 people from different Brazilian states, who were divided into working groups, Fasm held a general assembly on December 12 and 15 in which the following working groups were created and discussed: communication, coordination/working group, coordination of services, occupation, science, ethics and human rights, and general conference.

The participants of the general assembly included mental health service users and their families, mental health professionals, researchers and students from universities, activists advocating for the end of institutionalization, amongst others. On the first day, after the introductions, a panel session was held to analyze the broad context, including progressive politicians such as Erika Kokay, Glauber Braga, and Flávio Serafini. Also taking part were administrators, representatives from universities and human rights and mental health social movements, as well as service users and their families. After that, a session was held with the aforementioned working groups.

During the general assembly, a desire to forge a new social compact between the field of mental health and progressive democratic social movements was expressed. The latter groups include indigenous peoples, maroon communities, LGBTQI, the Landless Workers’ Movement (Movimento dos Trabalhadores Sem Terra), and the Homeless Workers’ Movement (Movimento dos Trabalhadores Sem Teto). The different discussions held resulted in a summarized report containing data on the encounter and the approved proposals (Fasm, 2020b).

On December 21, 2020, Fasm published volume 1 of its dossier. This document reproduced dozens of manifestos, releases, and petitions produced by different groups and entities from organized civil society against the changes to the national public mental health policy. The dossier was a response to the document produced by the Brazilian Association of Psychiatry and was presented at a meeting of the National Council of Secretaries of Health of the Ministry of Health (Fasm, 2020a).

The 257-page-long dossier is an important historical and political document on the work and engagement of social and political entities, presenting a broad overview of the struggle for the defense of mental health across the country, insofar as the different documents it brings together were signed by hundreds of individuals and entities (Fasm, 2020a).

Overall, the dossier compiled 73 documents by different entities including: Brazilian Association of Collective Health, Associação Brasileira de Saúde Mental (Brazilian Association of Mental Health), Associação Brasileira de Médicas e Médicos pela Democracia (Brazilian Association of Doctors for Democracy), Rede Nacional de Médicos Populares (National Network of Popular Medicine), and many more (Fasm, 2020a).

One of the documents was produced by Fasm itself, entitled “Fasm launch manifesto with petition,” which, according to Fasm, had already received 68,000 signatures by the date of publication. The manifesto expressly contradicts the terms of the Brazilian Association of Psychiatry’s “Guidelines…,” considering it “debatable from a technical and scientific perspective, insofar as it insists on adopting an outdated model that: dismantles the concept of comprehensive care and disregards the humanity of people with mental suffering, reducing them to the condition of mentally ill, and speaking in the name of the whole of psychiatry and the rest of society” (Fasm, 2020b, p.11). The manifesto disputes the representativeness of the Brazilian Association of Psychiatry vis-a-vis different segments of mental health and denounces its outdated scientific ethical and humanitarian stance in this field, stating:

We are the United Front for the Defense of Mental Health, Psychiatric Reform, and Anti-asylum Struggle and we state in the strongest terms that this is not the mental health policy we want. We are against mental asylums, and we restate our position in favor of care in freedom and the end of all psychiatric hospitals, therapeutic communities, and institutions of a similar nature, replacing them definitively with a network of open and community-based services (Fasm, 2020b, p.11).

Other manifestos stand out in the dossier, such as the Manifesto of Research Groups in Mental Health, Psychosocial Care, Psychiatric Reform and Related Areas, for the defense of the Mental Health Policy, which garnered 218 signatures (Fasm, 2020b, p.25). Alongside statements by Brazilian actors and groups, the dossier published texts supporting the fight against institutionalization by authorities from other countries, such as the Italian psychiatrist Benedetto Saraceno and the Spanish psychiatrist Manuel Desviat, from the movement for psychiatric reform in Spain.

The text by Saraceno was written in response to the document produced by the Brazilian Association of Psychiatry, which had misrepresented his own position. After listing six points deserving of investment in key areas of mental health, Benedetto Saraceno concludes:

A radical paradigm shift is needed from a biomedical model to a model based on the promotion and defense of human rights and capable of fostering interventions that have a real impact on the social determinants of disease, which are, for example, poverty, social exclusion, and low level of education.

This is why I identify fully with the principles and practices of the Brazilian fight against institutionalization and the reform experiments in previous years, which have been aligned with struggles for users’ rights and worked to put a complete and definitive end to psychiatric hospitals (Fasm, 2020b, p.256).

## Final considerations

The Jair Bolsonaro government was a material representation of state and institutional attacks on several sectors of everyday life. It cut funding for research by public universities, institutes, and facilities in both health in general and mental health in particular. Furthermore, in a context of exacerbated institutionalization, it invested in passing segregating and medicalizing policies that reinforced the stigmatization of individuals, promoted institutional violence, infringed human rights, and exacerbated class-, race-, and gender-based inequality.

Since 2016, the dismantling of psychosocial care in the public sector has been consolidated in Brazil to the benefit of private institutions, which are investing in an obsolete asylum model designed to attract public funding. In other words, there has been a shift away from the national public agenda and political project, which until then were channeling resources into strengthening and expanding community-based services. However, not only did this government exacerbate this scenario, but it used arbitrary and anti-democratic strategies, which included deliberately reducing the oversight of services and the transparency of mental health policies. These strategies also aimed to undermine social control and public participation in institutional spheres of power at a municipal, state, and national level.

Nonetheless, while state actors may have made a raft of retrograde moves, these actions have had the effect of rousing organized civil society. The current anti-institutionalization movement is embraced by a range of social actors, such as university-based research groups, associations of mental health service users and their relatives, professional entities, trade unions in general, public administrators, researchers, professors, students, and others. The creation of Fasm, in the first year of the pandemic, was a major move in the reaction against the dismantling of the psychiatric reform, with its uncompromising defense of care in freedom and the end of total institutions and reinforcement of open, community-based networks of services. The ultra-conservative national movement has not gone away, and in the current political climate, one of the key tenets of mental health concerns the construction of national, state, and municipal mental health conferences, providing an opportunity for political disputes to be played out on all levels and for the development of public policies for national mental health. This is a challenge for those currently engaged in the struggles and resistance and will have ramifications into the future.

For the historian and political activist Mike Davis, the pandemic only worsened the crisis of capitalism and its toxic legacy of austerity and budget cuts for healthcare, especially since the 2008 recession. For this reason, the way forward must be in the public arena, fighting against all kinds of violence and exploitation, from evictions of squatters to layoffs of workers. But according to him that is not enough: socialism must also be reinstated in political discourse as a project that goes beyond national demands. There is a pressing need for international solidarity (Davis et al., 2020).

In Brazil, the curbs on public spending and primary expenditure for 20 years, driven by the logic of contemporary capitalism under the influence of financial capital, should not be forgotten or neglected as a popular cause. In other words, the fiscal austerity law must be put back on the agendas of social movements as they mobilize resistance in daily life in general and in mental health in particular.

The challenges facing the progressive camp when it comes to the mental health agenda are not inconsiderable. They include the importance of creating ongoing dialogue with groups that have been historically overlooked and silenced, such as indigenous and peripheral populations and children orphaned in the pandemic, to mention just three. Policies must be reconstructed that envisage a comprehensive set of intersectoral actions in mental health, in coordination with service networks, designed to enable the delivery of care to people in freedom. As the ongoing struggle against authoritarianism proceeds, discussions must be held about the ways forward and the potential means of supporting people in the area of mental health and strengthening the care network for the most vulnerable. But above all, on a macropolitical level, there must be a resolute struggle for popular democracy, above all against the pervasiveness of neoliberal policies.

## Data Availability

They are not in a data repository.

## NOTE

1 All the meetings and coordination actions were conducted online in observance of the lockdown recommendation at the beginning of the pandemic issued by the World Health Organization.
